# Expression and Functions of Immediate Early Response Gene X-1 (IEX-1) in Rheumatoid Arthritis Synovial Fibroblasts

**DOI:** 10.1371/journal.pone.0164350

**Published:** 2016-10-13

**Authors:** Akio Morinobu, Shino Tanaka, Keisuke Nishimura, Soshi Takahashi, Goichi Kageyama, Yasushi Miura, Masahiro Kurosaka, Jun Saegusa, Shunichi Kumagai

**Affiliations:** 1 Department of Rheumatology and Clinical Immunology, Kobe University Graduate School of Medicine, 7-5-2 Kusunoki-cho, Chuo-ku, Kobe, 650-0017, Japan; 2 The Orthopedic Surgery, Kobe University Graduate School of Medicine, 7-5-2 Kusunoki-cho, Chuo-ku, Kobe, 650-0017, Japan; 3 Clinical Laboratory, Kobe University Hospital, 7-5-2 Kusunoki-cho, Chuo-ku, Kobe, 650-0017, Japan; 4 The Center for Rheumatic Diseases, Shinko Hospital, 1-4-47 Wakinohama-cho, Chuo-ku, Kobe, 651-0072, Japan; JAPAN

## Abstract

In rheumatoid arthritis (RA), synovial fibroblasts (RA-SFs) accumulate in affected joints, where they play roles in inflammation and joint destruction. RA-SFs exhibit tumor-like proliferation and are resistant to apoptosis. Although RA-SF activation is well described, negative regulators of RA-SF activation are unknown. We previously reported that histone deacetylase (HDAC) inhibitors facilitate apoptosis in RA-SFs. Here we found that RA-SFs treated with the HDAC inhibitor Trichostatin A (TSA) exhibited an upregulation of the immediate early response gene X-1 (IEX-1). IEX-1 has roles in apoptosis sensitivity, cell-cycle progression, and proliferation, and is reported to be involved in immune responses, inflammation, and tumorigenesis, and to have anti-arthritic properties. To investigate IEX-1’s role in RA-SFs, we used in vitro-cultured synovial fibroblasts from RA and osteoarthritis (OA) patients. We confirmed that TSA upregulated the IEX-1 protein and mRNA expressions in RA-SFs by western blotting and quantitative RT-PCR. Inhibiting HDAC1, 2, and 3 (but not 6 or 8) also upregulated IEX-1. The IEX-1 mRNA levels were higher in RA-SFs than in OA-SFs, and were further upregulated in RA-SFs by the pro-inflammatory cytokines TNFα and IL-1β. The staining of surgical specimens showed that IEX-1 was present in the pannus from affected RA joints. Si-RNA-mediated IEX-1 knockdown upregulated the lipopolysaccharide (LPS)-induced expression of TNFα and various chemokine mRNAs, indicating that IEX-1 downregulates TNFα and chemokines. Furthermore, apoptosis analysis showed that IEX-1 knockdown protected RA-SFs from apoptosis induced by TSA or by an anti-Fas mAb, indicating that IEX-1 is pro-apoptotic in RA-SFs. Collectively, our results showed that IEX-1 is induced by TNFα and IL-1β in RA-SFs, in which it suppresses TNFα and chemokine production and induces apoptosis; thus, IEX-1 negatively regulates RA-SF activation. Further investigation of IEX1’s functions in RA-SFs may lead to new therapeutic approaches for RA.

## Introduction

Rheumatoid arthritis (RA) is a systemic inflammatory disease that progressively destroys the joints [1]. Synovial hyperplasia, which occurs in regions of aggressive joint destruction and is composed of synovial fibroblasts (RA-SFs) and infiltrating lymphocytes and macrophages, is a characteristic of RA. In particular, RA-SFs are actively involved in persistent inflammation and joint destruction [2–4]. RA-SFs are characterized by increased cell survival and destruction of surrounding tissue, and play a pro-inflammatory role in immune responses.

RA-SFs have tumor-like proliferative properties and are resistant to apoptosis. This resistance to apoptosis may be related to somatic p53 mutations, the activation of the NF-κB pathway in RA-SFs, and the elevated expression of such anti-apoptotic molecules as Bcl-2, Fas-associated death domain-like interleukin-1β-converting enzyme-inhibitory protein (FLIP), and sentrin-1/small ubiquitin-like modifier (SUMO-1) [5]. We previously showed that histone deacetylase (HDAC) inhibitors facilitate apoptosis in RA-SFs in the presence of an anti-Fas mAb [6]. Thus, we used a differential display technique to search for genes that were up- or downregulated in RA-SFs by the HDAC inhibitor trichostatin A (TSA), and found that the immediate early response gene X-1 (IEX-1) was upregulated in TSA-treated RA-SFs (data not shown).

IEX-1, also known as IER3 (immediate early response 3) or p22/PRG1, is a 27-kDa glycosylated protein that has 156 amino acids and shares no significant sequence similarities with other proteins. IEX-1 is expressed in a broad range of human tissues and is upregulated by various stimuli, such as ionizing radiation or UV exposure, death receptor agonists, growth factors, viral infection, or biomechanical strain [7, 8]. Changes in IEX-1 expression alter cells’ sensitivity to apoptosis, their cell-cycle progression, and their proliferation rate. Recent clinical studies showed that IEX-1 is expressed in cancer specimens and may be a prognostic indicator for cancers, depending on the cell type. For example, the IEX-1 expression in tumor tissues may be associated with a better prognosis in pancreatic cancer [8].

Furthermore, studies in IEX-1 knockout mice showed that IEX-1 is involved in immune responses and inflammation, as well as in tumorigenesis [9–11]. Studies in IEX-1 deficient mice demonstrated that IEX-1 has anti-arthritic properties; one of the proposed mechanisms is enhanced Th17 differentiation through reactive oxygen species-mediated signaling [11].

To date, no role has been reported for IEX-1 in RA-SFs. Here we characterized IEX-1’s expression and function in RA-SFs, and showed that IEX-1 is highly expressed in RA-SFs and negatively regulates RA-SF activation.

## Materials and Methods

### Reagents

TSA was purchased from Sigma-Aldrich (St Louis, MO, USA), CI994 from Biovision (Milpitas, CA, USA), romidepsin (FK228) and RGFP966 from BPS Bioscience (San Diego, CA, USA), tubastatin from Focus Biomolecules (Plymouth Meeting, PA, USA), and PCI-34051 from Santa Cruz Biotechnology, Inc. (Dallas, Texas, USA). Anti-IEX-1 antibody was purchased from Santa Cruz Biotechnology Inc. and from Sigma-Aldrich. Anti-β-actin antibody was purchased from Sigma-Aldrich. Lipopolysaccharide (LPS), IL-1, TNFα, IL-17, IL-6, and PDGF were purchased from R&D Systems (Minneapolis, MN, USA). The anti-Fas mAb was from MBL Co. Ltd. (Nagoya, Japan).

### Cell culture

RA-SF and synovial specimens were obtained from patients who fulfilled the American College of Rheumatology 1987 criteria for RA and who underwent joint-replacement surgery. SF and synovial samples were also collected from patients with osteoarthritis. All patients provided written consent prior to participating in this study, in accordance with the World Medical Association Declaration of Helsinki Ethical Principles for Medical Research Involving Human Subjects. The study protocol, including consent procedures, was approved by the Ethics Committee of the Kobe University Graduate School of Medicine.

The collected tissues were minced and incubated with 4 mg/ml collagenase and then with 0.05% trypsin (Difco, Detroit, MI, USA). The isolated cells were maintained in Dulbecco’s Modified Eagle’s Medium (DMEM) supplemented with 10% fetal calf serum, streptomycin/penicillin, and non-essential amino acids. Adherent cells after 3–5 passages were used as RA-SFs for experiments [6] [12].

### siRNA

IEX-1 was knocked down using HiPerfect Transfection Reagent (Qiagen, Valencia, CA, USA) according to the manufacturer’s protocol. Briefly, cells were seeded into 24-well plates (1 × 10^5^ cells/well) and grown for 3 h. Anti-IEX-1 siRNA (37.5 ng/well) (Qiagen) or scrambled siRNA (Qiagen) and 3 μl of HiPerfect Transfection Reagent were dissolved in 100 μl/well of serum-free DMEM, incubated for 10 min at room temperature, and added to the wells. After a 48-h transfection time, the cells were used for various assays[12].

### Apoptosis assay

Cell viability was assessed using WST-8 (Dojindo, Kumamoto, Japan), as described previously [6] [12]. Briefly, siRNA-treated cells were plated in 96-well plates (3 × 10^4^ cells/well) for 24 h, and were then incubated in the presence of TSA and/or anti-Fas mAb for 18 h. The samples were then pulsed with WST-8 for 3–4 h, and the optical density was measured at 450 nm using a microplate reader (Bio-Rad, Hercules, CA, USA) to determine cell viability. Each experiment was performed using quadruplicate cultures.

Apoptotic cell death was quantified with the Cell Death Detection ELISA (Roche Diagnostics, Mannheim, Germany), which specifically detects mono- and oligonucleosomes in the cytoplasm, following the manufacturer’s protocol. In other experiments, cells were incubated in the dark for 15 min with Annexin V FITC and propidium iodide (PI) using an Annexin-V-FLUOS Staining Kit (Roche Diagnostics). Apoptotic cells were detected as annexin V–positive cells using a FACScan (Beckton Dickinson, San Jose, CA, USA) [6] [12].

### Western blotting

Cells were collected and lysed in lysis buffer consisting of 50 mM Tris-HCl (pH 7.5), 150 mM NaCl, 1 mM EDTA, 1% NP40, 1 mM PMSF, 1 mM NaF, 1 mM NaVO_4,_ and a protease inhibitor mix (Roche). Protein concentration was measured using BCA Protein Assay Reagents (Pierce, Rockford, IL, USA). Equal amounts of cell lysate were separated by sodium dodecyl sulfate-polyacrylamide gel electrophoresis, and then transferred to a PVDF membrane (Millipore, Natick, MA, USA). The membrane was blocked with blocking buffer (5% skim milk in 1% Tween 20 in Tris-based saline) and then incubated with primary antibodies. Next, the membrane was incubated with an HRP-conjugated secondary antibody, and immunoreactive bands were visualized using ECL Western Blotting Detection Reagents (Amersham) [6] [12].

### Quantitative RT-PCR

The mRNA levels of IEX-1 and other molecules were quantified by real-time reverse transcription-polymerase chain reaction (RT-PCR). Total RNA was isolated using the RNeasy Kit (Qiagen), and 1 μg RNA was reverse-transcribed into cDNA using a QuantiTect Reverse Transcription Kit (Qiagen). The primer pairs were purchased from Qiagen. Real-time PCR was performed using a Pico Real 96-well system (Thermo Fisher Scientific, Waltham, MA). All primers were purchased from Qiagen. The mRNA expression levels were expressed as a ratio to that of G3PD mRNA in the same sample.

### Immunohistochemistry

Paraffinized sections of synovium from RA and OA patients were deparaffinized and rehydrated by successive washes with xylene and graded ethanol. Antigens were retrieved with HistoVT One (Nacalai Tesque, Inc., Tokyo, Japan). Endogenous peroxidase was blocked with 0.3% hydrogen peroxide in methanol. The slides were then incubated with normal serum for 1hour at room temperature and then with the primary anti-IEX-1 antibody (Sigma-Aldrich) or with rabbit polyclonal IgG (Abcam) overnight at 4°C. The slides were incubated with a biotinylated secondary antibody for 30 min, followed by avidin and biotinylated horseradish peroxidase (Santa Cruz Biotechnology, Inc., CA, USA) for immunohistochemical staining.

## Statistical Analysis

Results are expressed as the mean ± SEM. All data represent results from four or more independent experiments. Mann-Whitney tests were applied to compare between 2 groups. For comparison between paired samples, paired *t*-tests was applied. When 3 or more groups were compared, analysis of variance (ANOVA) was applied and followed by either Tukey Method or Mann-Whitney tests. Results were considered significant if the 2-sided *P* value was less than 0.05.

## Results

### TSA upregulates IEX-1 in RA-SFs

**Fig 1:**
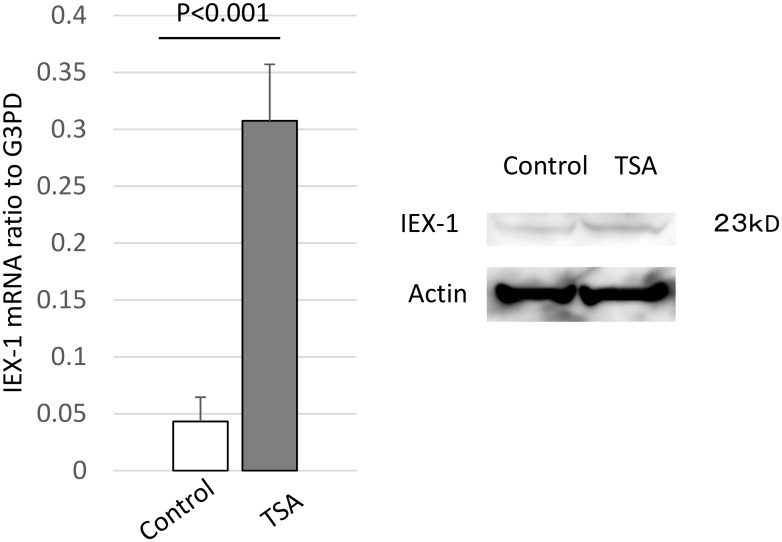
TSA upregulates IEX-1 in RA-SFs. SFs from 5 RA patients were stimulated with TSA (1 μg/ml) or vehicle for 24 h, and IEX-1 was measured by quantitative RT-PCR (left) and western blotting (right).

We previously reported that inhibiting HDACs induces apoptosis in RA-SFs. To clarify the mechanism of the HDAC inhibitor TSA, we used a differential display technique to search for potential candidate genes regulated by TSA, and found that that TSA upregulated IEX-1 (data not shown). We used real-time RT-PCR and western blotting to confirm that TSA upregulated IEX-1 mRNA (Fig 1).

### HDAC1, 2, and 3 (but not 6 or 8) induce IEX-1

**Fig 2:**
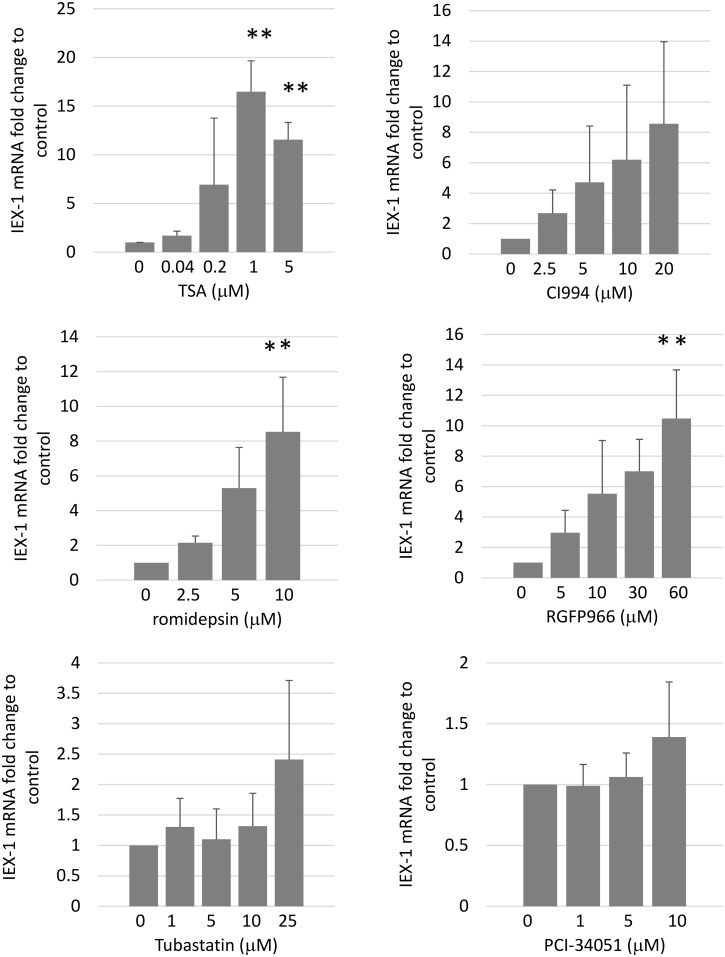
IEX-1 is upregulated by certain HDAC inhibitors. RA-SFs were stimulated with the HDAC inhibitors TSA (HDACs1-10), CI994 (HDAC1), romidepsin (HDAC1, 2), RGFP966 (HDAC3), tubastatin (HDAC6), or PCI-34051 (HDAC8) for 24 h at the indicated doses, and the IEX-1 expression was measured by quantitative RT-PCR. The means ± SD are shown from 4 experiments from different patients. ANOVA was applied and followed by Tukey Method; *P<0.05, **P<0.01.

The HDACs consist of 18 enzymes grouped into four classes (I-IV). TSA suppresses class I HDACs (1, 2, 3, and 8) and class II HDACs (4, 5, 6, 7, 9, and 10) [13]. We used specific HDAC inhibitors to determine which HDAC upregulates IEX-1 in RA-SFs. TSA suppresses HDACs1-10; CI994 suppresses HDAC1, romidepsin suppresses HDAC1 and 2, RGFP966 suppresses HDAC3, tubastatin suppresses HDAC6, and PCI-34051 suppresses HDAC8[13]. As shown in Fig 2, TSA, romidepsin, and RGFP966 induced IEX-1 mRNA according to dosage. CI994 did not upregulated IEX-1 mRNA (P<0.1). Neither tubastatin nor PCI-34051 upregulated IEX-1 mRNA at all. Thus, the inhibition of both HDAC1 and 2, or HDAC 3, but not HDAC6 or 8, could be involved in the upregulation of IEX-1 expression in RA-SFs.

### IEX-1 mRNA expression is higher in RA than OA SFs

**Fig 3:**
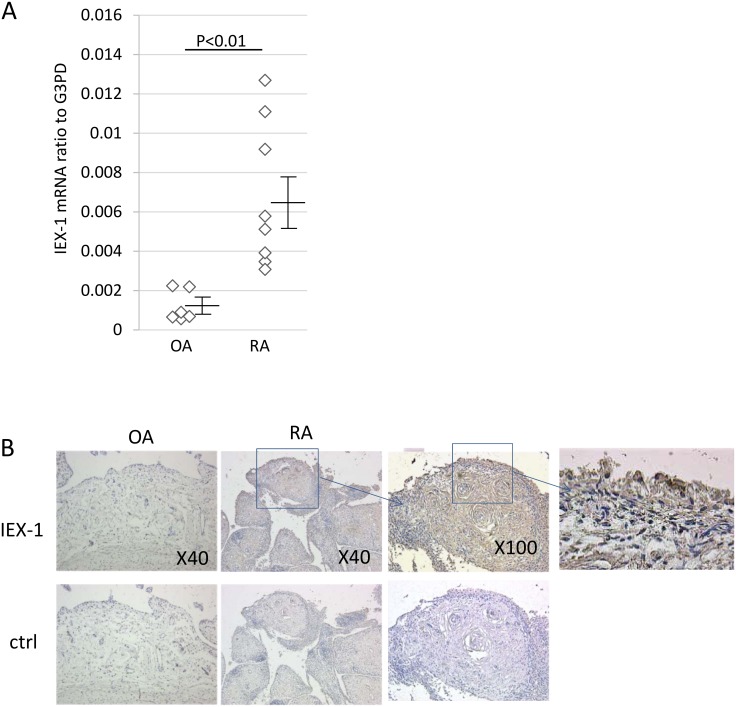
IEX-1 is expressed in RA-SFs. SFs from 6 OA and 8 RA patients were grown in vitro. (A) IEX-1 mRNA was measured by quantitative RT-PCR. (B) Immunohistochemical analysis of the IEX-1 expression in RA and OA synovium; data shown are representative of three RA and two OA patients.

To determine whether IEX-1 mRNA plays a specific role in RA-SFs, we compared the IEX-1 mRNA levels in cultured RA-SFs and OA-SFs using quantitative RT-PCR, and found that the RA-SFs expressed higher levels of IEX-1 mRNA (Fig 3A). We then stained the IEX-1 protein in synovial specimens from RA and OA patients (Fig 3B). IEX-1 was present in the pannus from affected RA joints.

### TNFα and IL-1β strongly induce IEX-1 mRNA in RA-SFs

**Fig 4:**
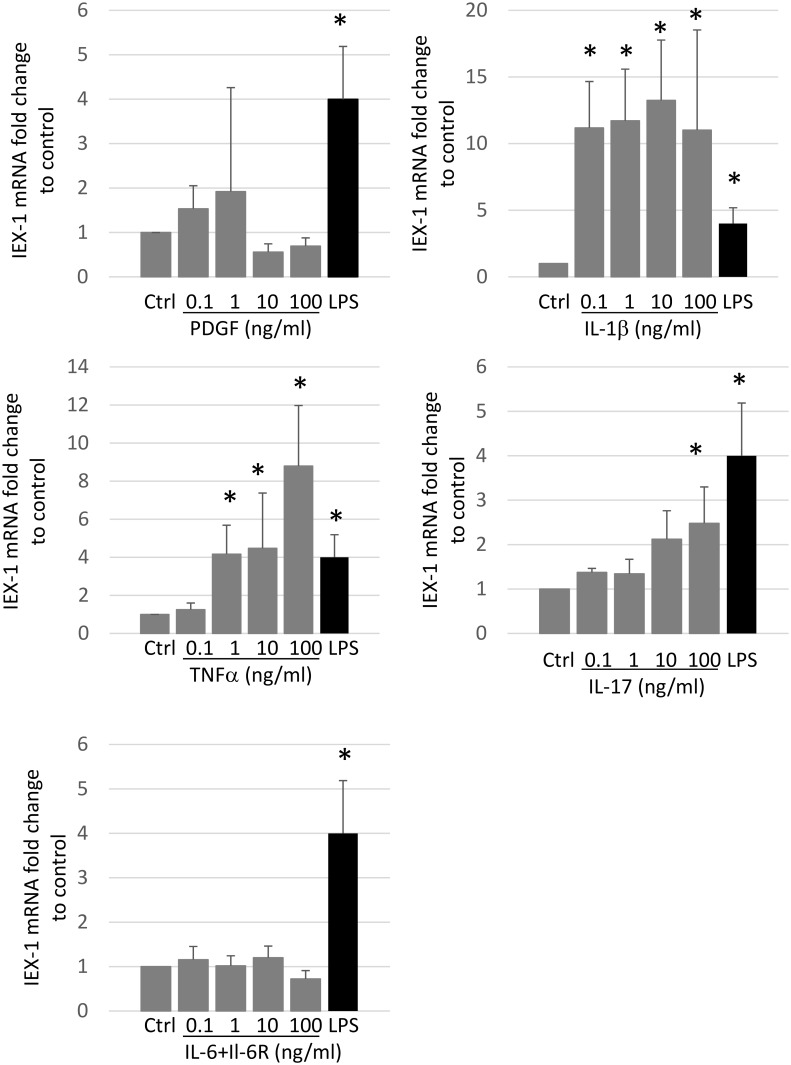
TNFα and IL-1β induce IEX-1 in RA-SFs. RA-SFs were stimulated with LPS (1 μg/ml) or the indicated doses of PDGF, IL-1β, TNFα, IL-17, or IL-6 and IL-6R for 24 h. IEX-1 mRNA levels were measured using quantitative RT-PCR. The means ± SD are shown for 3–4 experiments with different RA-SFs. ANOVA was applied (ANOVA) was applied and followed by either Tukey Method or MannWhitney test for IL-1β; *P<0.05.

Next, to identify stimuli that induce IEX-1 in RA-SFs, we stimulated RA-SFs with LPS, IL-1β, TNFα, IL-17, IL-6, or PDGF for 18 h and measured the effect on IEX-1 mRNA expression. TNFα and IL-1β upregulated the IEX-1 mRNA in RA-SFs, but IL-6 and PDGF did not (Fig 4). IL-17 also up-regulated IEX-1 mRNA to a lesser extent than IL-1β and TNFα. We therefore speculated that TNFα and IL-1β upregulate IEX-1 under inflammatory conditions, leading to the higher IEX-1 expression in RA-SFs.

### IEX-1 suppresses TNFα and chemokine mRNAs

**Fig 5:**
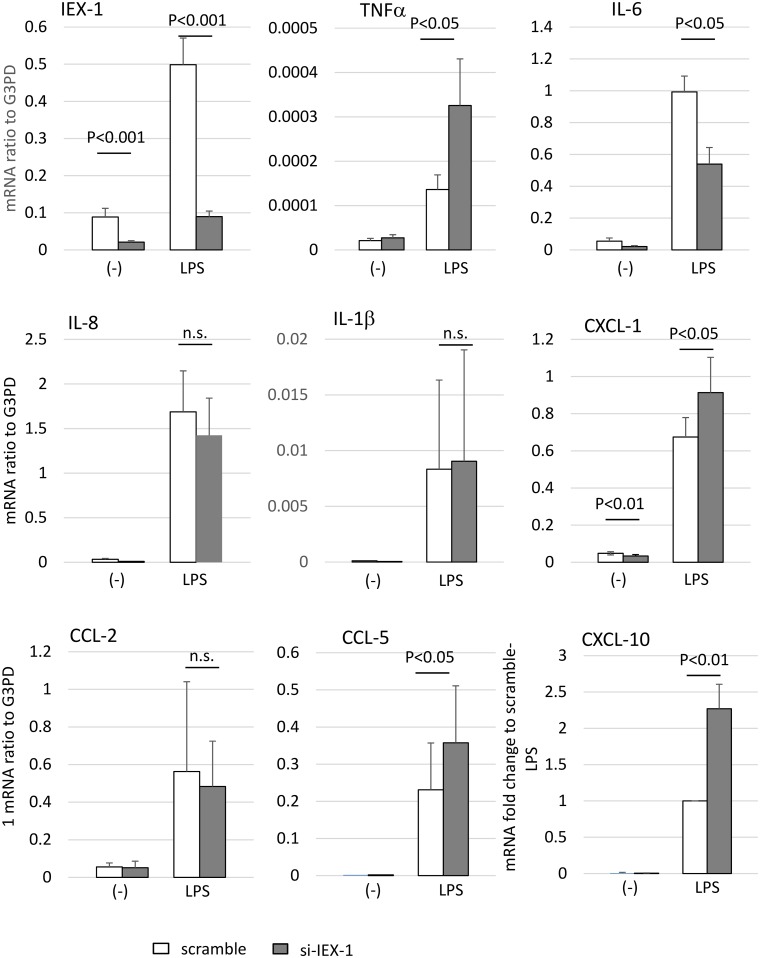
IEX-1 affects RA-SF cytokine and chemokine production. IEX-1 was knocked down in RA-SF samples using siRNA, after which the cells were stimulated with LPS (1 μg/ml) for 16 h. Cytokine and chemokine mRNAs were measured by quantitative RT-PCR, and the results were expressed as a ratio to the G3PD mRNA. The CXCL-10 results were expressed as the fold-change compared to LPS-stimulated, scrambled siRNA–transfected cells. The means ± SD are shown for 7 experiments with different RA-SFs. Statistical significance was determined by a paired Student’s *t-*test.

To elucidate the role of IEX-1 in RA-SFs, we transfected RA-SFs with siRNA against IEX-1 and measured the effect on cytokine and chemokine production (Fig 5). The cells were transfected with scrambled or IEX-1 siRNA for 48 h and stimulated with 1 μg/ml LPS for 16 h, after which they were assayed for various cytokine and chemokine mRNAs. The CXCL-10 mRNA level differed greatly between patient samples, so the CXCL-10 mRNA level was expressed as a fold-change compared to the level in LPS-stimulated, scrambled siRNA–transfected cells. The IEX-1 mRNA was successfully knocked down in RA-SFs. In RA-SFs transfected with scrambled siRNA, LPS induced the mRNA expression of TNFα, IL-1β, IL-6, IL-8, CXCL-1, CCL-5, CCl-2, and CXCL-10. When IEX-1 was knocked down, the mRNA levels of TNFα, CXCL-1, CCL-5, and CXCL-10 were significantly upregulated compared to those in scrambled siRNA–transfected cells, indicating that IEX-1 suppresses the production of these molecules. In contrast, IEX-1 siRNA suppressed the LPS-induced IL-6 expression. Thus, IEX-1 regulates and primarily suppresses the production of cytokines and chemokines by RA-SFs.

### IEX-1 promotes apoptosis mediated by TSA + anti-Fas mAb

We previously reported that TSA facilitates apoptosis mediated by the anti-Fas mAb in RA-SFs [6]. Given that TSA induces IEX-1, we hypothesized that IEX-1 is involved in this TSA/anti-Fas-mediated apoptosis. We examined the effect of IEX-1 on this type of apoptosis by knocking down IEX-1 in RA-SFs using siRNA and treating the cells with TSA (1 μM) and/or anti-Fas mAb (500 ng/ml). We assayed the viability of these cells with WST-8 and found that TSA and anti-Fas mAb treatment synergistically induced cell death, as we previously reported [6].

**Fig 6:**
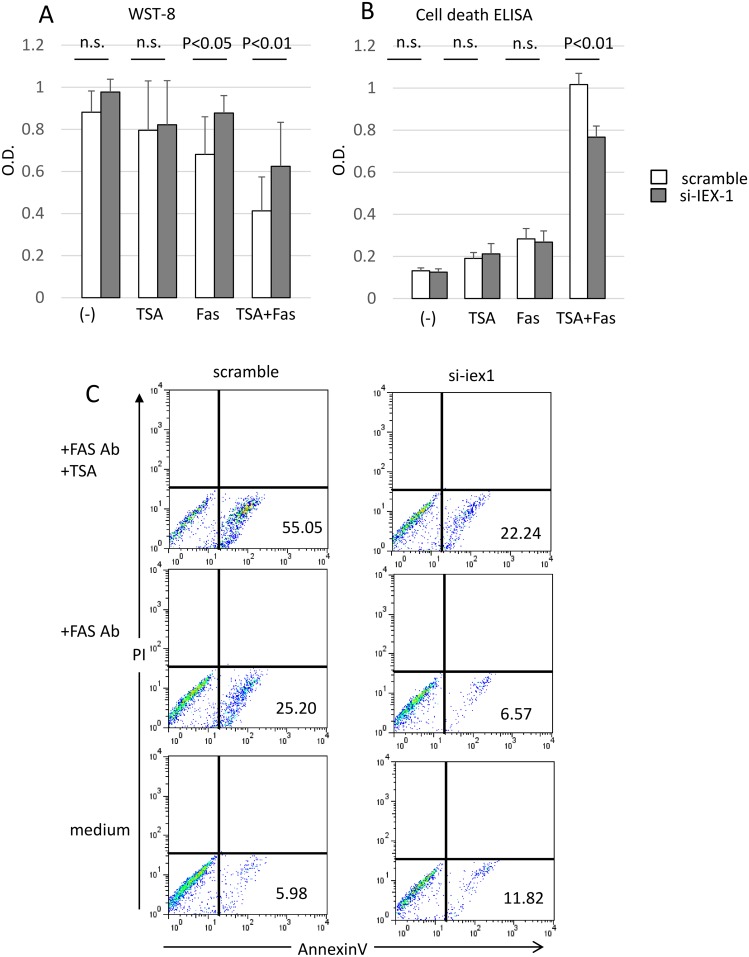
IEX-1 promotes TSA- and Fas-induced apoptosis in RA-SFs. IEX-1 was knocked down in RA-SFs using siRNA, and the cells were seeded in 96-well plates and incubated with TSA (1 μM) and/or anti-Fas mAb (500 ng/ml) for 18 h. (A) Viable cells were quantified with a WST-8 assay. Results are expressed as O.D. The means ± SD are shown for 5 experiments with different RA-SFs. Statistical significance was analyzed by a paired Student’s *t-*test. (B) Apoptosis was measured with a Cell Death Detection ELISA; results shown are representative of 3 experiments. (C) FACS analysis of annexin V–positive cells. Data shown are representative of 3 experiments with similar results.

IEX-1 knockdown significantly augmented cell survival in the presence of the anti-Fas mAb alone or in combination with TSA (Fig 6A). An ELISA to detect cell death showed that the increased cell counts resulting from silencing IEX-1 were mediated by the inhibition of apoptosis (Fig 6B). FACS analysis of annexin V on the cell surface, which marks apoptotic cells, also showed that knocking down IEX-1 significantly reduced apoptosis in the presence of the anti-Fas mAb alone or in combination with TSA (Fig 6C). These results indicated that IEX-1 has pro-apoptotic effects in RA-SFs, particularly in the presence of the anti-Fas mAb and HDAC inhibitors.

### TNFα and IL-1β also induce IEX-1 mRNA in OA-SFs

**Fig 7:**
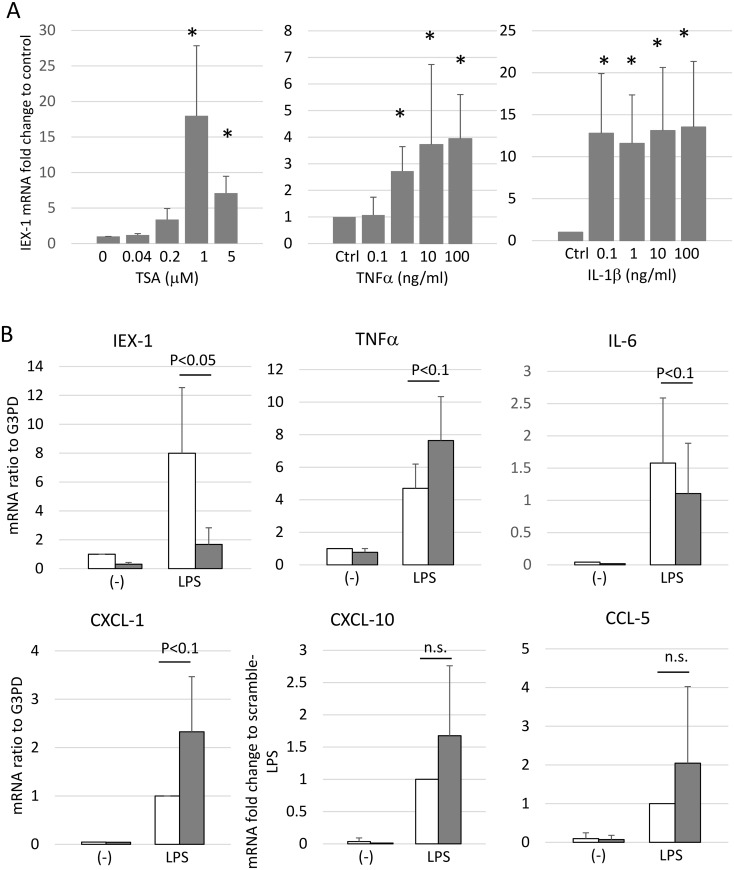
IEX-1 is also induced in OA-SF and affects its cytokine and chemokine production. (A) OA-SFs were stimulated with the indicated doses of TSA, TNFα, or IL-1β for 24 h. IEX-1 mRNA levels were measured using quantitative RT-PCR. The means ± SD are shown for 5 experiments with different OA-SFs. Statistical significance was determined by ANOVA followed by Tukey Method; *P<0.05. (B) IEX-1 was knocked down in OA-SF samples using siRNA, after which the cells were stimulated with LPS (1 μg/ml) for 16 h. Cytokine and chemokine mRNAs were measured by quantitative RT-PCR, and the results were expressed as a ratio to the G3PD mRNA. The CXCL-10 and CCL-5 results were expressed as the fold-change compared to LPS-stimulated, scrambled siRNA–transfected cells. The means ± SD are shown for 4 experiments with different OA-SFs. Statistical significance was determined by a paired Student’s *t-*test.

Next, we examined whether the regulation and function of IEX-1 is similar or different in OA-SF compared to RA-SF. As shown in Fig 7A, TSA, TNFα and IL-1β induced IEX-1 mRNA in OA-SF to the similar extent to that in RA-SF, suggesting that HDACi potentially induce IEX-1 in synovial fibroblasts irrespective of disease type.

To further elucidate roles for IEX-1 in OA-SF, the effects of IEX-1 on LPS-induced cytokine and chemokine mRNA expression were determined using siRNA, in the same protocol done in RA-SF. The IEX-1 mRNA was also well knocked down by siRNA. LPS stimulation also strongly induced TNFα, IL-6, CXCL-1, CXCL-10 and CCL-5. Interestingly, when IEX-1 is knocked down, the mRNA levels of TNFα, CXCL-1, CXCL-10 and CCL-5 tended to increase and that of IL-6 tended to decrease. These trends were exactly the same as those observed in RA-SF, although they were not statistically significant. Thus we speculate that the effects of IEX-1 on cytokine and chemokine production is similar between RA-SF and OA-SF.

### IEX-1 did not suppress apoptosis mediated by TSA + anti-Fas mAb in OA-SF

**Fig 8:**
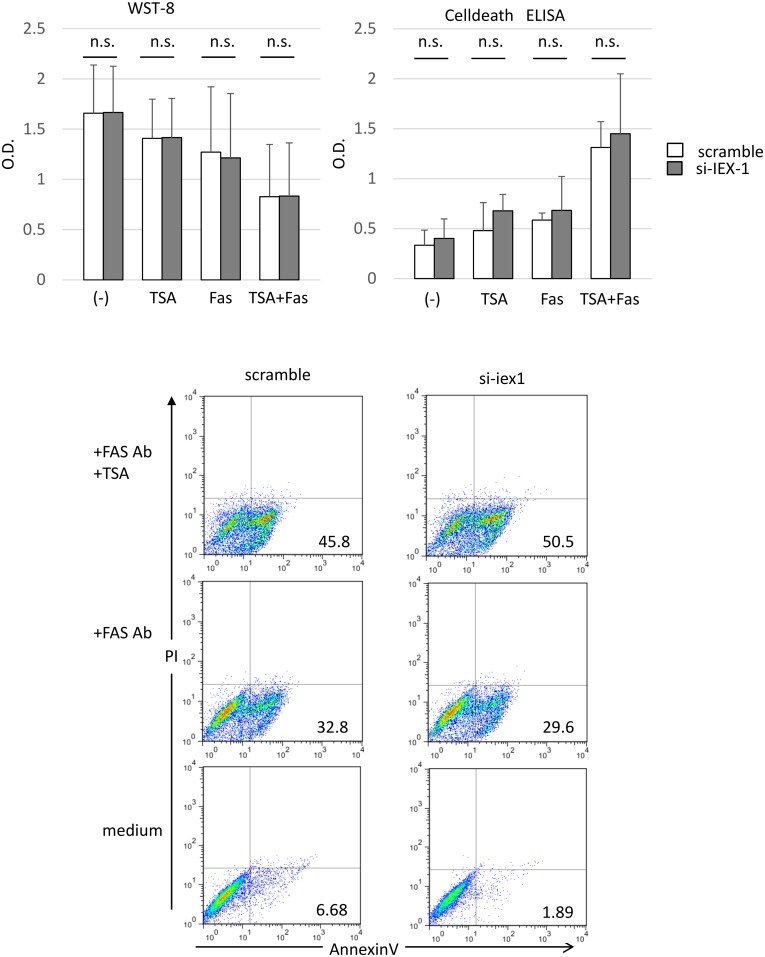
IEX-1 does not affect TSA- and Fas-induced apoptosis in OA-SFs. IEX-1 was knocked down in OA-SFs using siRNA, and the cells were seeded in 96-well plates and incubated with TSA (1 μM) and/or anti-Fas mAb (500 ng/ml) for 18 h. (A) Viable cells were quantified with a WST-8 assay. Results are expressed as O.D. The means ± SD are shown for 3 experiments with different OA-SFs. Statistical significance was analyzed by a paired Student’s *t-*test. (B) Apoptosis was measured with a Cell Death Detection ELISA. The means ± SD are shown for 3 experiments with different OA-SFs. Statistical significance was analyzed by a paired Student’s *t-*test. (C) FACS analysis of annexin V–positive cells. Data shown are representative of 3 experiments with similar results.

Finally we examined the effect of IEX-1 on apoptosis mediated by TSA+anti-Fas mAb by knocking down IEX-1 in OA-SFs with the same protocol in Fig 6. We assayed the viability of these cells with WST-8, and determined apoptotic cell death with ELISA and FACS analysis of annexin V (Fig 8). We found that TSA and anti-Fas mAb treatment synergistically induced cell death in OA-SFs. However, IEX-1 knockdown did not augmented cell survival in the presence of the anti-Fas mAb alone or in combination with TSA (Fig 8). These results indicated that IEX-1 has no effects of apoptosis induced in the presence of the anti-Fas mAb and HDAC inhibitors in OA-SFs, suggesting that the roles for IEX-1 in apoptosis are different between RA-SF and OA-SF.

## Discussion

RA-SFs have an aggressive phenotype and actively participate in the RA inflammatory process. RA-SFs are thought to be initially activated by inflammatory cytokines, and then to become persistently active independently of cytokines [4]. Although the RA-SF activation process is well documented, negative regulators of RA-SF activation have not been identified. We here show that IEX-1 suppresses RA-SF activation by inducing apoptosis and modulating cytokine and chemokine production. Since IEX-1 is upregulated by TNFα and IL-1β, IEX-1 may function as a negative regulator of inflammation during the initiation phase of RA pathogenesis. In addition, cultured RA-SFs expressed more IEX-1 than did OA-SFs, indicating that RA-SFs in the persistently active phase may constitutively express IEX-1.

Our results showed that IEX-1 is induced in RA-SFs by TNFα, IL-1β, and IL-17, but not by IL-6, indicating that these cytokines play different roles in RA pathology. The IEX-1 promoter is controlled by several transcription factors, including NF-κB, AP1, AP2, VD3R, RAR, Sp1, c-Myc, and p53 [7]. Among them, NF-κB may be involved in IEX-1 induction by TNFα and IL-1β because these cytokines activate NF-κB and NF-κB play an important role in inducing IEX-1 [14]. IEX-1 is also induced by γ-radiation, UV exposure, death receptor agonists, growth factors, viral infection, and biomechanical strain. This study is the first to demonstrate that HDAC inhibitors, particularly those that suppress class I HDACs, induce IEX-1. This finding is compatible with our previous data showing that HDAC1 and 2 are elevated in RA-SFs [12].

RA-SFs promote persistent inflammation and joint destruction by producing various mediators. Inflammatory cytokines, including TNFα and IL-6, sustain inflammation. Chemokines such as CCL-5 and CXCL-1 attract leukocytes, and matrix metalloproteinases (MMPs) and RANKL help destroy cartilage and bone [4]. We have shown that IEX-1 negatively regulates TNFα, which induces inflammation, and the inflammatory chemokines CCL-5 and CXCL-1, which recruit lymphocytes and other inflammatory cells. Thus, our results indicate that IEX-1 plays an anti-inflammatory role in the pathogenesis of RA. On the other hand, IEX-1 did not affect the expression of MMP1, MMP3, or MMP13 mRNA in our experiments (data not shown).

In the present study, we found that IEX-1 promotes anti-Fas mAb–mediated apoptosis in RA-SFs, showing that IEX-1 is pro-apoptotic in these cells. In this respect, we previously reported that TSA cooperates with anti-Fas mAb–mediated apoptosis in RA-SFs, and we here show that IEX-1 is induced by TSA and promotes apoptosis in RA-SFs. Taken together, our data demonstrate that the apoptosis induced by anti-Fas mAb and TSA in RA-SFs may be mediated, at least in part, by the induction of IEX-1. The role of IEX-1 in apoptosis varies by the type of cell and stimulus [8]. IEX-1 exerts a range effects on cells, including suppressing NF-κB inhibition, sustaining MAPK activation, and controlling ROS production [15] [16]. The nuclear localization of IEX-1 is also important for its pro-apoptotic activity [17]. However, we found that IEX-1 had no effect on expression of Bcl-2, Bcl-XL, MCL-1, FLIP, Bim, BID, Bad, Bax, Bak, NOXA, or PUMA (data not shown). How IEX-1 promotes apoptosis in RA-SFs remains to be elucidated.

Interestingly, regulation and function of IEX-1 in OA-SF were similar to that in RA-SF. TSA, TNFα and IL-1β 1induced IEX-1 mRNA in OA-SF, and IEX-1 modulated cytokine and chemokine mRNA expression in the same way as in RA-SF as shown in Fig 7. However, apoptosis induced in OA-SF by TSA plus anti-Fas mAb was not affected by knockdown of IEX-1 (Fig 8), indicating that IEX-1 plays a role in RA-SF but not in OA-SF. The difference in apoptois might be explained by lower levels of IEX-1 mRNA in OA. However, we think it difficult to explain the difference between RA-SF and OA-SF by the IEX-1 alone. Further work is required.

The anti-arthritic effect of IEX-1 demonstrated in IEX-1–deficient mice has been ascribed to enhanced Th17 differentiation [11]. Our data demonstrate that IEX-1 also acts against arthritis by suppressing RA-SFs, indicating that IEX-1 exerts its anti-arthritic properties through multiple mechanisms. This is the first report showing that HDAC inhibitors strongly upregulate IEX-1. HDAC inhibitors suppress arthritis and other types of inflammation, presumably through several mechanisms [18]. HDAC inhibitors upregulate IEX-1, and IEX-1 suppresses RA-SF; thus, IEX-1 may play a role in anti-arthritic effects of HDC inhibitors.

In summary, IEX-1 is highly expressed in cultured RA-SFs and negatively regulates RA-SF function and survival, and inhibiting HDACs induces IEX-1 in RA-SFs. Thus, deepening our understanding of IEX-1 functions in RA-SFs may lead to new therapeutic approaches for RA.

## Conclusion

IEX-1 is highly expressed in cultured RA-SFs and is induced by TNFα. IEX-1 suppresses TNFα production and induces apoptosis in RA-SFs. Thus, IEX-1 appears to negatively regulate RA-SF activation.

## Abbreviations

IEX-1: Immediate early response gene X-1
RA-SF: rheumatoid arthritis synovial fibroblasts
RA: rheumatoid arthritis
OA: osteoarthritis
TSA: Trichostatin A
FLIP: Fas-associated death domain-like interleukin-1β-converting enzyme-inhibitory protein
SUMO-1: sentrin-1/small ubiquitin-like modifier
HDAC: histone deacetylase
MMPs: matrix metalloproteinases
